# Cytokinin biosynthesis genes expressed during nodule organogenesis are directly regulated by the KNOX3 protein in *Medicago truncatula*

**DOI:** 10.1371/journal.pone.0232352

**Published:** 2020-04-30

**Authors:** Mahboobeh Azarakhsh, Andrey M. Rumyantsev, Maria A. Lebedeva, Lyudmila A. Lutova

**Affiliations:** 1 Department of Genetics and Biotechnology, Saint Petersburg State University, Saint Petersburg, Russia; 2 Cell and Molecular Biology Department, Kosar University of Bojnord, Bojnord, North Khorasan Province, Iran; Estacion Experimental del Zaidin - CSIC, SPAIN

## Abstract

Cytokinin is an important regulator of symbiotic nodule development. Recently, KNOTTED1-LIKE HOMEOBOX 3 transcription factor (TF) was shown to regulate symbiotic nodule development possibly via the activation of cytokinin biosynthesis genes. However, the direct interaction between the KNOX3 TF and its target genes has not been investigated up to date. Here, using EMSA analysis and SPR-based assay, we found that MtKNOX3 homeodomain directly binds to the regulatory sequences of the *MtLOG1*, *MtLOG2*, and *MtIPT3* genes involved in nodulation in *Medicago truncatula*. Moreover, we showed that *MtLOG2* and *MtIPT3* expression patterns partially overlap with *MtKNOX3* expression in developing nodules as it was shown by promoter:GUS analysis. Our data suggest that MtKNOX3 TF may directly activate the *MtLOG1*, *MtLOG2*, and *MtIPT3* genes during nodulation thereby increasing cytokinin biosynthesis in developing nodules.

## Introduction

KNOX (KNOTTED-like homeobox) homeodomain transcription factors (TFs) are important regulators of plant development. In land plants, there are two classes of KNOX genes, class I and class II [1]. Class I KNOX TFs regulate shoot apical meristem (SAM) development and maintenance, and the modulation of their expression patterns in SAM is associated with leaf shape diversity. The role of class II KNOX TFs is less understood. Class II KNOX genes are broadly expressed in differentiating tissues and mature organs [2]. Among them, *Arabidopsis* KNAT7 TF has been shown to regulate secondary cell wall biosynthesis [3], whereas KNAT3 was reported to regulate abscisic acid responses during germination [4]. In *Arabidopsis* loss-of-function mutations in class II KNOX genes resulted in impaired differentiation of aerial organs and highly complex leaves, the phenotype observed in gain-of-function mutants of class I KNOX genes. Based on these and other findings, it was suggested that class II KNOX TFs have antagonistic function relative to class I KNOXs in plant development [5].

In SAM, class I KNOX TFs act through the activation of cytokinin biosynthesis genes *ISOPENTENYL TRANSFERASEs (IPTs)* in *Arabidopsis* [6, 7]. Recently, it was shown that class I KNOX TF also activates the expression of cytokinin biosynthesis via an *ISOPENTENYL TRANSFERASE* gene in bryophyte *Physcomitrella patens*, suggesting that KNOX-cytokinin regulatory module predated the origin of indeterminate meristem in vascular plants [8].

In contrast to class I KNOX TF, the mechanisms of class II KNOX function and their target genes have not been well-established. Previously, we found that class II TF KNOX3 regulates the development of symbiotic nodule in *Medicago truncatula* and pea [9]. Spontaneous nodule-like structures were formed on transgenic roots with *MtKNOX3/PsKNOX3* overexpression, where the increased expression levels of cytokinin biosynthesis and cytokinin response genes were observed. Moreover, *MtKNOX3* knockdown via RNA-interference resulted in downregulation of cytokinin biosynthesis genes *ISOPENTENYLTRANSFERASE 3 (MtIPT3)* and *LONELY GUY 2 (MtLOG2)* in developing nodules of *M*. *truncatula *[9]. Recently, it was shown that expression of cytokinin biosynthesis genes of *LOG*s and *IPT*s families under constitutive promoter resulted in spontaneous nodule formation in *Lotus japonicus* [10]. These data support our suggestion that the KNOX3 TF acts through the activation of cytokinin biosynthesis genes of *LOG* and *IPT* families during nodulation, since KNOX3 overexpression also led to spontaneous nodule formation. The results obtained later by Di Giacomo et al. who studied the role of the *KNAT3/4/5*-like genes, namely *MtKNOX3*, *MtKNOX5*, *MtKNOX9*, and *MtKNOX10*, in nodule development are partially in agreement with our data: simultaneous down-regulation of *MtKNAT3/4/5*-like genes resulted in the decreased expression of cytokinin response gene *MtRR4* [11]. However, in this study both *MtKNOX3* overexpression and silencing of the *MtKNAT3/4/5‐*like genes resulted in the increased formation of fused nodule, a phenotype previously observed in *efd* (ethylene response factor required for nodule differentiation) mutant [12], and the expression of the *MtEFD* gene was also decreased in the transgenic roots with RNAi of *Mt KNAT3*/*4*/*5‐like* genes according to Di Giacomo et al. [11]. Given the fact that MtEFD was shown to activate the *MtRR4* gene [12], the Di Giacomo et al. suggested that Mt KNAT3/4/5‐like TFs act upstream of the *EFD*/*RR4* regulatory module [11].

Here, we found that class II KNOX TF, MtKNOX3, directly regulates cytokinin biosynthesis genes of *IPT* and *LOG* families. Our data suggest that a member of class II KNOX, MtKNOX3, is involved in the activation of cytokinin biosynthesis during nodule development, as it was previously shown for class I KNOX TFs in SAM. Therefore, we demonstrated that class II KNOX TF can be also involved in KNOX-cytokinin regulatory module, and it is recruited to regulate nodule development in legume plants.

## Materials and methods

### Molecular cloning

The *MtIPT3* promoter (including 2768 bp upstream of the MtIPT3 coding sequence) were amplified using high fidelity Phusion polymerase (Thermo Fisher Scientific, USA) with attB sites (ubderlined) added to the forward and reverce primers (pMtIPT3-Forward primer: AAAAAAGCAGGCTTCCAAAATCAAAATAGAAGAACA; pMtIPT3-Reverce primer: CAAGAAAGCTGGGTTGATGATGATAAAAATTAATTCA). Amplified fragment first was cloned into the pDONR221 vector (Invitrogen, USA), and then inserted into the pBGFWS7.0 vector containing the β-glucuronidase (GUS) reporter gene and 35S terminator sequence using LR clonase enzyme (Invitrogen, USA). The pMtLOG2::GUS construct was kindly provided by Sofie Goormachtig (Ghent University, VIB, Gent, Belgium).

### *Agrobacterium rhizogenes*-mediated plant transformation and rhizobial inoculation

*M*. *truncatula* A17 seeds were surface sterilized with concentrated sulfuric acid, then washed 7–8 times with sterile distilled water and transferred to the plates with 1 percent agar. Plates were held in refrigerator on +4°C for one day, and after that seeds were germinated at the room temperature in a dark place during 48 hours. Germinated seedlings were sectioned about 5 mm lower than hypocotyl and were infected by *A*. *rhizogenes* strain MSU440 containing *pMtIPT3*::*GUS* and *pMtLOG2*::*GUS* constructs. The infected seedlings were placed on Fahraeus medium [13] for 5 days without antibiotic in the growth chambers under a 16-h photoperiod at 21°C (75% relative humidity). Subsequently, plants were transferred to Emergence medium [14] containing 300 mg/ml cefotaxime and were grown on this medium for three weeks. Plants were transferred to a fresh Emergence medium each week. After 3 weeks, plants were placed in vermiculite containing pots with Fahraeus medium, and were inoculated in 7 days with *Sinorhizobium meliloti* strain Sm2011. *S*. *meliloti* strain Sm2011 was cultivated up to OD600 of about 0.7, in YEB liquid medium [15], and 1 ml of the suspension culture was used for inoculation of each plant.

### Histochemical localization of GUS activity

Analysis of promoter activity was performed using the reporter gene GUS (β-glucuronidase). The transgenic roots with nodules were harvested in NT buffer (100 mM Tris/50mM NaCl) and then transferred to the GUS staining buffer (NT buffer: 100 mM Tris / 50 mM NaCl; 1.9 mM K3Fe(CN)_6_; 2.5 mM X-Gluc (5-bromo-4-chloro-3-indolyl glucuronide, Sigma-Aldrich, United States, pre-diluted in DMSO)) and were vacuum infiltrated for 15–20 minutes. GUS buffer was refreshed and the roots were put at 37°C until blue coloring. After staining the roots were infiltrated in PFA-GA fixative (3% paraformaldehyde, 0.5% GA in 1/3 MTSB buffer with 0.2% Tween-20 and 0.2% Triton X-100, 10% DMSO, pH 6.8) three times each for 5–7 minutes, fixed overnight at 4°C, and rinsed with 1/3 MTSB two times. The roots were then transferred to the 10, 20, 30, 40, 50, 60% ethanol in turn, each for one hour and 70% ethanol overnight after which the ethanol percent was decreased up to 10%. The roots were hold in TBS buffer (50 mM Tris-Cl, pH 7.6; 150 mM NaCl).

After fixation, roots and nodules were embedded in 3% agarose, and vibratome (Leica VT1200S) was used to prepare sections with 50μM thickness. The photos were taken using an inverted fluorescent microscope (Leica DMI6000).

The duration of GUS staining for *pMtKNOX3*::*GUS* and *pMtIPT3*::*GUS* was about 1 and 3 hours, respectively. For *pMtLOG2*::*GUS*, overnight staining was performed, however in mature nodules the signal was also detected after 6 hour staining. The whole-mount views of GUS-staining nodules were analyzed for all the nodules formed on transgenic roots of about 15–20 plants from each biological repeat and about 20 nodules were sectioned from each biological repeat. Three biological repeats were performed.

### Heterologous expression of MtKNOX3 in *Pichia pastoris* and protein extraction

The homeobox sequence of the MtKNOX3 gene (corresponding to amino acids 359 to 426 from the CDS of MtKNOX3), as well as the full-length coding region of MtKNOX3 (439 amino acids) were amplified using Phusion polymerase (Thermo Fisher Scientific, USA). The EcoRI restriction site (gaattc) was added to the forward primer sequence, and the XbaI restriction site (tctaga) was added to the reverse primer. (HD-For: aggaattcattttacgcaagagacgagc; HD-Rev: gctctagagcagttgaaggattgctgtgc; KNOX3-CDS-For: aggaattcatggcttaccaaaaccaac; KNOX3-CDS-Rev: gctctagagcgttttgagaccttttgcgtttg). The resulting fragments were cloned first into the pJET intermediate vector, and then inserted into the pPICZαA vector (Thermo Fisher Scientific, USA), which contains *Saccharomyces cerevisiae* alfa-MF sequence which is needed for the secretion of recombinant protein, C-myc epitope and also His tag, which is needed for the purification of protein. The constructs then were introduced into the yeast *Pichia pastoris* strain X-33. *P*. *pastoris* cells were grown in BMGY medium (1% yeast extract, 2% peptone, 100 mM potassium phosphate buffer, pH 6.0 1.34% YNB (yeast nitrogen base, Sigma), 4 × 10^−5^% biotin, 1% glycerin) for 48 hours. The cells were centrifuged (5000 rpm, 10 min.) and transferred to BMMY medium (1% yeast extract, 2% peptone, 100 mM potassium phosphate buffer, pH 6.0 1.34% YNB, 4 × 10^−5^% biotin, 0.5% methanol). Protein synthesis was induced using methanol for 72 hours. Then the cells were centrifuged (5000 rpm, 10 min) and the medium containing the protein was concentrated using Amicon Ultra-15 filters (Merck, cat. No. UFC901008). Isolation and purification of the MtKNOX3 homeodomain was performed using Ni-NTA agarose (QIAGEN, Ni-NTA Spin kit).

### Electrophoretic mobility shift assay

40bp biotinylated DNA sequences were synthesized and used for this experiment (http://www.biobeagle.com/ru). The annealing reaction was carried out in a total volume of 50 μl containing 2X annealing buffer (20mM TrisOH, 100mM NaCl, 2mM EDTA) in a thermal cycler (98°C for 5 min, then the temperature was decreased from 97°C to 24°C (in each step, the temperature was decreased by 1°C and for one minute), then the temperature was maintained at 24°C for 30 minutes and was held at 4°C). 10 fmol / μl of biotinylated double-stranded DNA was incubated with protein using the LightShift Chemiluminescent EMSA Kit (Thermo Scientific, 20148) in an EMSA binding buffer (100mM Tris, 500mM KCl, 10mM DTT; pH 7.5) with Poly dI- dC (1μg / μL in 10mM Tris, 1mM EDTA, pH 7.5) and 50% Glycerol. The double stranded DNA and protein were incubated for one hour at room temperature. For competition assay, unlabeled double-stranded DNA was added in 500, 1000 and 2000-fold excess compared to labeled DNA. Binding reactions were separated on a 10% polyacrylamide gel (acrylamide/bis-acrylamide, 29: 1) containing 3% glycerol in 0.5X Tris-borate-EDTA running buffer. After electrophoresis, DNA was transferred to the membrane (Biodyne B Nylon Membrane, 8cm × 12cm, 0.4μm pore size, Thermo Scientific, USA) using Mini Trans-Blot cell (BioRad, USA). Biotinylated DNA on the membrane was detected by chemiluminescence (Chemiluminescent Nucleic Acid Detection Module Kit, Thermo Scientific, USA) using a GeneGnome instrument (SynGene, India). In the case of mutated sequences, which were used as a control, TGAC motif was converted to the TCGC (according to the previous studies, GA nucleotides in the motif are the most important nucleotides for the interaction with KNOX proteins [16]).

### Surface plasmon resonance assay

The interactions between biotinylated double stranded oligonucleotides (ligand) and purified protein (analyte: MtKNOX3 homeodomain) were evaluated using surface plasmon resonance technology using the ProteOn XPR36 (Bio-Rad) available in the Resource Center for Molecular and Cell Technologies of St. Petersburg State University. The biotinylated double-stranded oligonucleotides with a concentration of 0.5–1μM in PBS (150 mM NaCl, 2 mM Na_2_HPO_4_) / Tween (pH 7.4) were immobilized on a sensor microchip that was coated with neutravidin (ProteOn NLC sensor chip, Bio-Rad) at a flow rate of 30μl / min. The interactions were evaluated at different protein concentrations (0, 1, 3, 5, 7, and 10 μM, the protein was diluted in PBS / Tween). PBS / Tween was used as the running buffer. As a competitor, an unlabeled double-stranded poly A-T sequence was added to the protein solution in 10-30-fold excess compared to the immobilized oligonucleotides. To prevent non-specific binding, BSA (Sigma Aldrich, UK) with a concentration of 670 μg / ml was also added to the protein solution. The data were obtained using ProteOn software (ProteOn manager software), and kinetic analysis was performed using the Langmuir estimation model [17, 18].

## Results

### The expression pattern of *MtKNOX3* partially overlaps with the expression patterns of its putative targets (*MtLOG2* and *MtIPT3*)

In our previous works, we showed that down regulation of *MtKNOX3* expression by RNAi resulted in a decreased expression of cytokinin biosynthesis genes *MtLOG2* and *MtIPT3*, as well as cytokinin response regulator gene (*MtRR4*) in developing nodules of *M*. *truncatula* [9]. Based on this observation we proposed that MtKNOX3 could be responsible for the activation of cytokinin biosynthesis genes *MtLOG2* and *MtIPT3* during nodulation.

**Fig 1:**
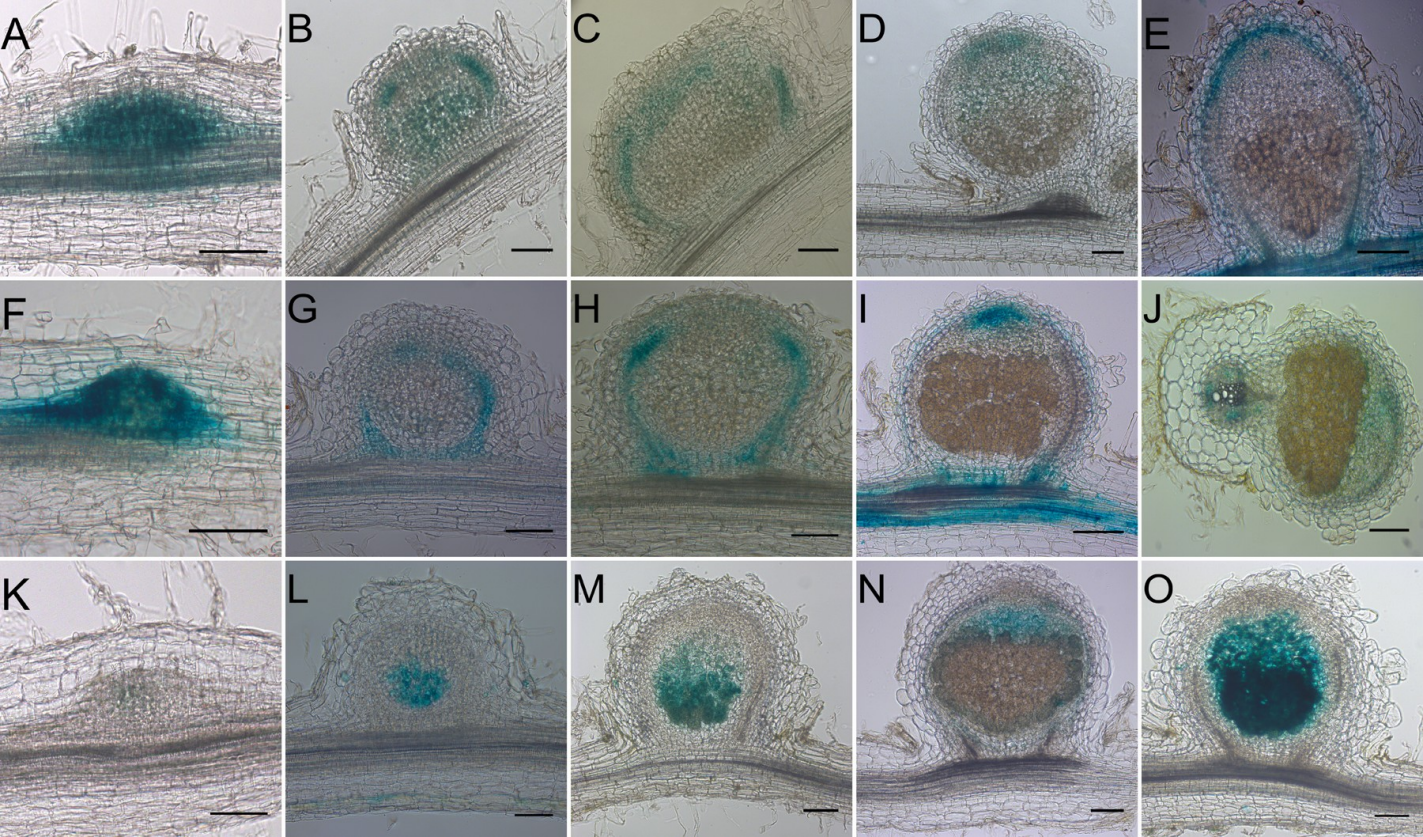
Tissue specific expression of *pMtKNOX3*:*GUS* (A-E), *pMtIPT3*:*GUS* (F-J), and *pMtLOG2*:*GUS* (K-O) at different stages of nodule development (nodule primordia at 3 dpi (A, F, K), developing nodules at 7 dpi (B, G, L), developing nodules at 12 dpi (C, H, M) and mature nodules at 18–21 dpi (D-E, I-J, N-O)). Bars, 100 μm. Thickness of sections, 50 μm.

To prove that *MtLOG2* and *MtIPT3* can be the targets of KNOX3 TF we first checked if these genes coexpress with the *MtKNOX3* gene in nodules. According to the *M*. *truncatula* LCM-RNA-seq data (https://iant.toulouse.inra.fr/symbimics/) [19], *MtKNOX3*, *MtLOG2*, and *MtIPT3* expression partially overlaps in different zones of the mature nodule (**S1 Fig**). To estimate the expression patterns of *MtKNOX3*, *MtLOG2*, and *MtIPT3* genes in more detail at different stages of nodule development, we further analysed the expression of these genes using promoter:GUS assay. Previously, the *pMtKNOX3*:*GUS* activity was visualized in developing nodules [9, 11]. The promoter of the *MtKNOX3* gene is active throughout the emerging nodule primordia at 3 dpi (days after inoculation) (**Fig 1A**) and throughout proliferating cells in developing nodule in the central part of the nodule as well as at its periphery at 7dpi (**Fig 1B**), whereas at later stages (12 dpi), *pMtKNOX3*:*GUS* activity is observed in provascular bundles and in the apical part of the nodule (**Fig 1C**; see also **Fig 2** in [9] and **Fig 8** in [11])). In mature nodules, *MtKNOX3* promoter activity was detected at the periphery region (nodule cortex in the apical part of the nodule) (**Fig 1E**), and in some nodules the faint *pMtKNOX3*:*GUS* activity was also detectable at the meristematic region (**Fig 1D and 1E**).

In this study, we analyzed the localization of *MtIPT3* and *MtLOG2* expression using promoter-GUS assay. In a way similar to *pMtKNOX3*::*GUS*, *pMtIPT3*:*GUS* activity was first detected throughout the emerging nodule primordia at 3 dpi (**Fig 1F)**, whereas at about 7 dpi the expression was observed at the periphery of proliferating cells in developing nodule (**Fig 1G**), and at the later stages of nodule development (12 dpi), the expression was restricted to the developing vascular bundle tissue (**Fig 1H**). In mature nodules, the expression was observed in the apical part of the nodule (**Fig 1I and 1J**), the region corresponding to the ends of vascular bundles and nodule meristem cells (**Fig 1I**), and in some nodules the faint GUS staining was also detected in the cells of infection zone (**Fig 1J**). In addition, the expression of *MtIPT3* was visualized in the vascular tissues of the main root (see longitudinal and cross-section of the nodule in **Fig 1I and 1J**, respectively) and in the lateral roots (**S2 Fig**).

In contrast to *pMtKNOX3*::*GUS* and *pMtIPT3*::*GUS* expression, the expression of *pMtLOG2*:*GUS* fusion was not detected in nodule primordia of 3 dpi (**Fig 1K**) and its expression was firstly observed in the center of emerging nodule primordia at about 7 dpi (**Fig 1L**) after overnight GUS staining. At 12 dpi, the expression was still detected in the center of developing nodule (**Fig 1M**), while in mature nodules, *pMtLOG2*:*GUS* activity was detected in the nodule infection zone after 6 hour of GUS staining (**Fig 1N**), and after overnight GUS staining the signal was also observed in the nodule fixation zone (**Fig 1O**). No *pMtLOG2*:*GUS* activity in the roots was found even after overnight staining.

### Regulatory sequences of the *MtIPT* and *MtLOG* genes contain conserved KNOX-binding motif

**Fig 2:**
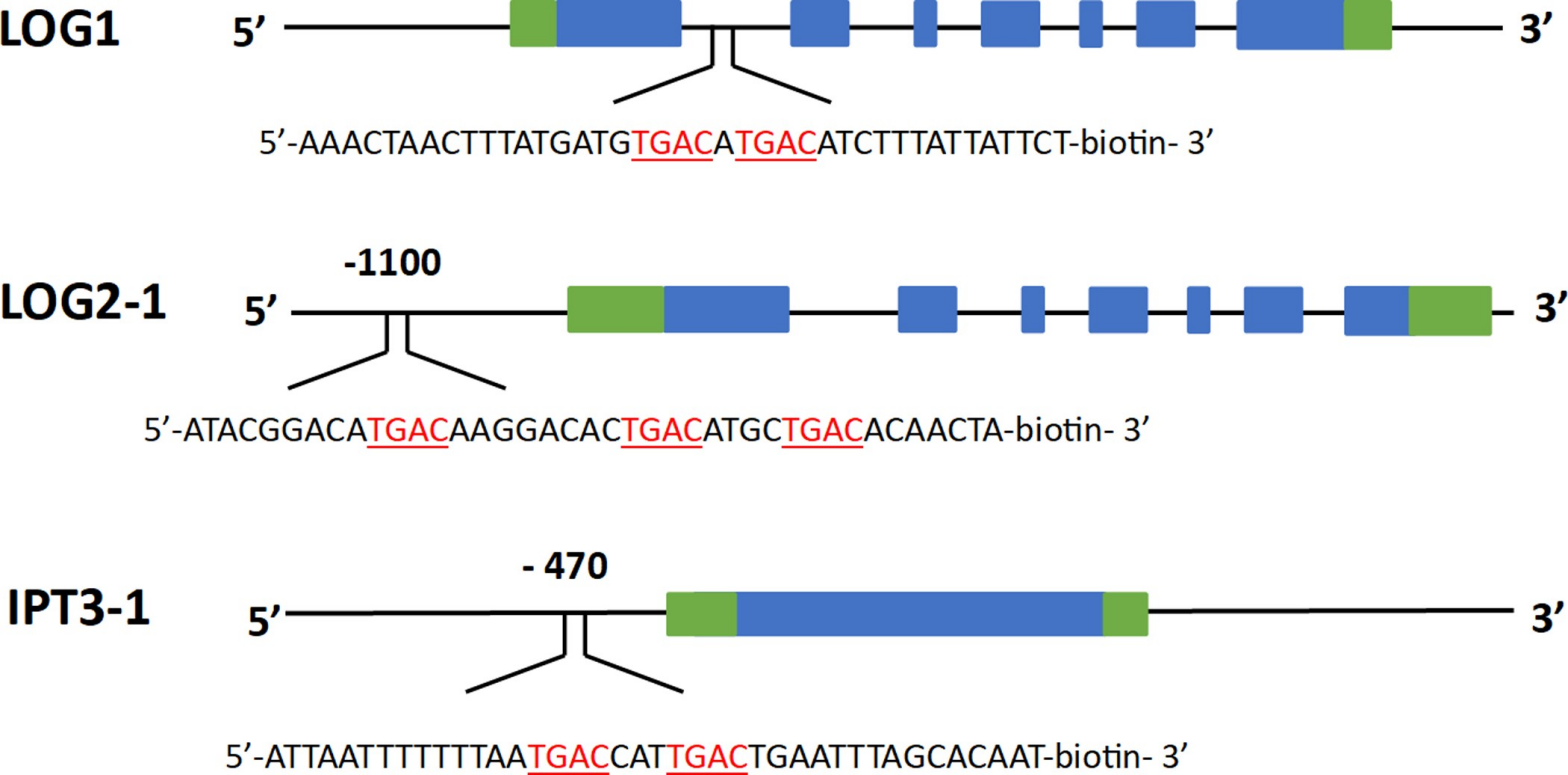
The localization of the putative MtKNOX3-binding sites in the regulatory sequences of the *MtLOG1*, *MtLOG2*, and *MtIPT3* genes. Exons are shown as blue blocks, while introns are represented by blue lines; 5’UTRs and 3’UTRs are shown as green blocks.

To understand if cytokinin biosynthesis genes can be the direct targets of MtKNOX3 TF, we searched for the possible *cis*-regulatory elements of KNOX family in the promoters and introns of the *MtLOG* and *MtIPT* genes. Previous studies in maize, potato and barley have shown that transcription factors of KNOX family preferentially bind to their target sites through a *cis*-regulatory element containing more than two TGAC motifs separated by 0 to 10 residues [20, 21, 22]. It has been shown that the homeodomain of maize KN1 binds with a much higher affinity to DNA sequences containing two TGAC motifs rather than one motif, both *in vivo* and *in vitro*. Therefore, the true KNOX binding sites should contain more than one TGAC motifs [16]. We searched for TGAC motif in the promoters and the introns of the putative target genes (*MtIPT*s and *MtLOGs*) and found the sites containing two TGAC motifs alongside in the promoter of the *MtLOG2* and *MtIPT3* genes, as well as in the first intron of the *MtLOG1* gene. Fig 2 shows a schematic representation of exon-intron structures of the *MtLOG1*, *MtLOG2*, and *MtIPT3* genes and the localization of the putative target sites (**Fig 2**).

To study the direct interaction of the MtKNOX3 TF with the regulatory sequences of the putative target genes, the protein was expressed in *P*. *pastoris* strain X-33. This eukaryotic system allows secretion of properly folded and post-translationally processed recombinant protein to the culture, which facilitates its isolation and purification [23]. Both full length CDS and homeodomain-encoding sequence of MtKNOX3 were cloned and transformed into *P*. *pastoris* cells (see Material and methods), but only MtKNOX3 homeodomain was secreted to the culture. It was isolated, purified and used for the subsequent experiments (see **S3 Fig**). Dot blot analysis showed that full length protein was present in small amounts in the cell lysates, but not in the culture medium (data was not shown). It is possible that full length CDS of MtKNOX3 contains sequences that prevent its secretion by *P*. *pastoris* cells. Therefore, the purified MtKNOX3 homeodomain was used for the protein-DNA interaction analysis.

### MtKNOX3 transcription factor binds to the regulatory sequences of *MtLOGs* and *MtIPT* gene *in vitro*

To evaluate whether MtKNOX3 TF can directly activate the expression of *MtLOG*s and *MtIPT*s, we used two different techniques: Electrophoretic Mobility Shift Assay (EMSA) and Surface Plasmon Resonance (SPR) based study of protein-DNA interactions.

**Fig 3:**
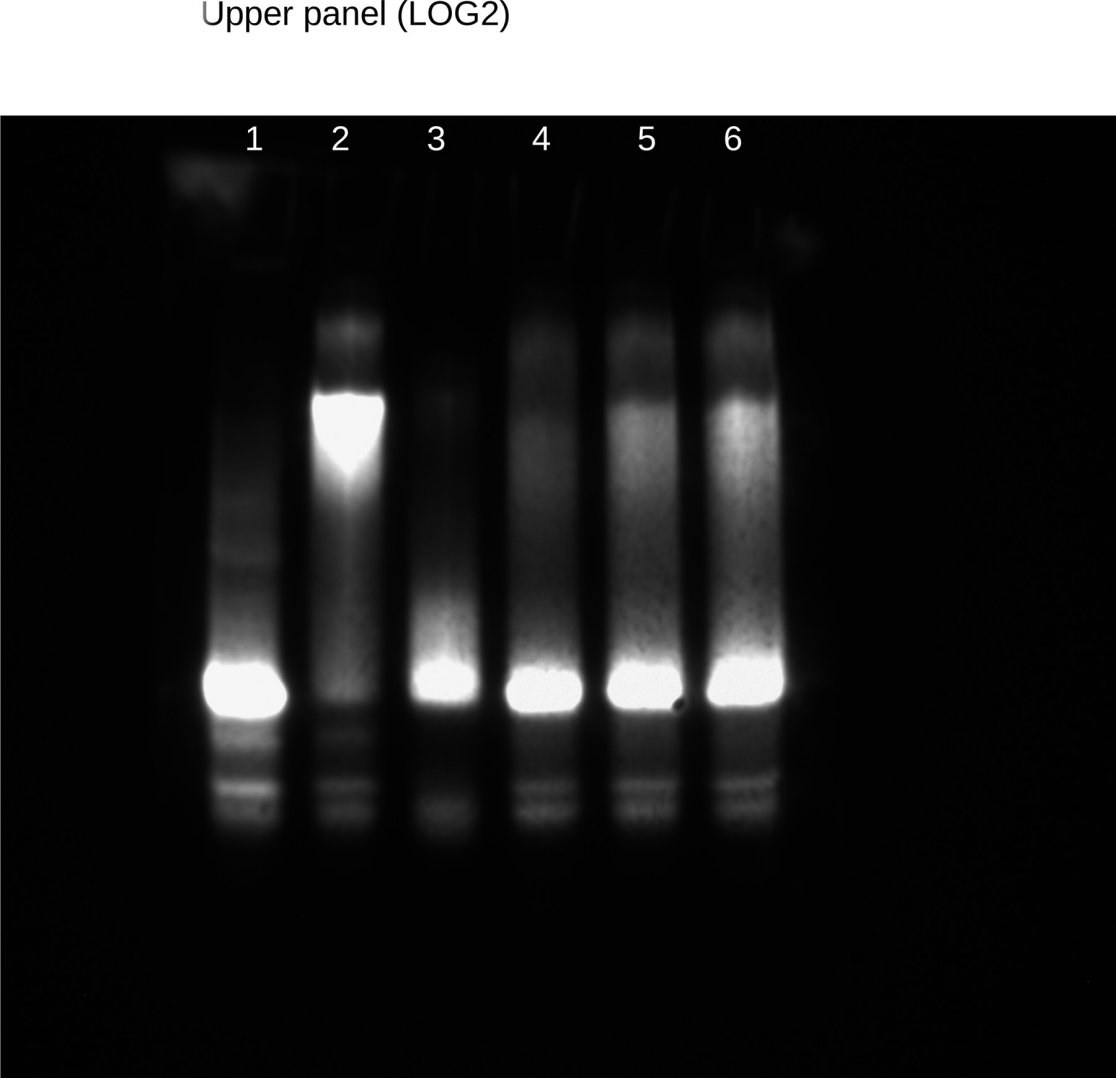
The results of EMSA experiment for the analysis of interaction between the homeodomain of MtKNOX3 and the regulatory sequences of the *MtLOG2*, *MtIPT3*, and *MtLOG1* genes. 1- free ds-DNA, 2- ds-DNA with the protein, 3- mutated ds-DNA with the protein, 4- ds-DNA with the protein and 2000X of competitor DNA, 5- ds-DNA with the protein and 1000X of competitor DNA, 6- ds-DNA with the protein and 500X of competitor DNA. The protein amount is the same in all wells (1000 ng).

Using EMSA we showed that MtKNOX3 homeodomain binds to the regulatory sequences of *MtLOG2*, *MtLOG1*, and *MtIPT3* genes, containing two or more TGAC motifs, while the mutated sequences (TGAC converted to the TCGC) were unable to be bound by the MtKNOX3 homeodomain at the same protein concentration (**Fig 3**). Even in the presence of competitor dsDNA (500, 1000 fold excess of competitor DNA, which has the same sequences but was unlabeled), the interaction was still observed (**Fig 3**).

The interaction of MtKNOX3 homeodomain with the regulatory sequences was also confirmed using SPR-based protein-DNA interaction assay (**S4 Fig**). Previously, this approach has been successfully used to study direct interaction between transcription factors and their target DNA sequences [24]. Poly A-T sequence, which was used as a negative control, showed no interaction with KNOX3 homeodomain at the same concentrations of protein (**S5 Fig**). **Table 1**shows the kinetic and affinity of binding reaction between MtKNOX3 homeodomain and putative regulatory sequences of the *MtLOG1*, *MtLOG2*, and *MIPT3* genes. High association constants (K_a_) and low dissociation constants (K_d_) show the high affinity of binding reactions.

**Table 1:** Binding kinetics and affinity of interactions between MtKNOX3 homeodomain and regulatory sequences of *MtLOG2*, *MtIPT3* and *MtLOG1*.

Sequence	K_a_ (1/Ms)	K_d_ (1/s)	KD (M)	Rmax (RU)	Chi2 (RU)
**LOG1**	2.95E+05	1.64E-01	5.55E-07	342.37	30.19
**LOG2-1**	3.46E+03	9.70E-02	2.80E-05	328.24	21.84
**IPT3-1**	2.26E+05	5.03E-01	2.22E-06	87.86	5.56
**polyA-T (control)**	3.56E-05	4.47E+01	1.26E+06	1.45E-02	12.09

For comparison, the data for the negative control (poly A-T sequence) are presented. K_a_—association constant, K_d_ -dissociation constant, KD-equilibrium constant, RU-response units.

Therefore, we showed that MtKNOX3 protein can directly bind to the cis-regulatory elements of *MtLOG2*, *MtIPT3*, and *MtLOG1* genes in two different experiment conditions, while the control DNA (poly A-T sequence) showed no interaction in both experiment conditions (**S5 Fig**). These results indicate that MtKNOX3 TF can be responsible for the activation of cytokinin biosynthesis genes through direct interaction with their regulatory sequences.

## Discussion

Previously, we found that RNAi-mediated down-regulation of *MtKNOX3* resulted in a decreased expression of cytokinin biosynthesis genes *MtLOG2* and *MtIPT3* [9]. These genes are activated in nodulating roots according to our data [9] and the data obtained by Mortier et al. [24]. Here, we investigated the spatial expression pattern of these genes in developing nodules.

The spatial expression pattern of the *MtIPT3* gene has not been investigated previously. Using *pMtIPT3*:*GUS* fusion we found that this gene is expressed in the developing nodule primordium, at later stages (at 12 dpi) its expression was observed in the vascular bundles, and in mature nodules (18–21 dpi) *pMtIPT3*:*GUS* was active in the apical part of the nodule.

The expression pattern of *MtLOG*s genes during nodulation has been studied previously by promoter:GUS analysis [25]. According to Mortier et al., *pMtLOG1*:*GUS* exhibited more intense activity which was detected in the nodule primordium, and at later stages–at the nodule apex, whereas only faint GUS staining was observed in the center of developing nodules for the *pMtLOG2*:*GUS* construct [25]. In our work to visualize the expression of *MtLOG2* gene we used the same construct. In the nodule primordium, *pMtLOG2*:*GUS* activity was visible in the central zone as it was found by Mortier et al.; however, in mature nodules, we observed intense GUS activity in the nodule infection zone as well as in the nodule fixation zone after more prolonged GUS staining.

According to our results, the expression of *MtIPT3* overlaps with *MtKNOX3* at early stages (3 dpi), while at this stage *MtLOG2* shows no expression (see **Fig 1A, 1F and 1K**). Later, the expression of *MtIPT3* is observed in the periphery of developing nodule primordia at 7 dpi and the expression of *MtLOG2* is firstly observed at this stage in the center of developing nodule. At this stage of nodule development (7 dpi) both *MtIPT3* and *MtLOG2* show overlapping expression with the *MtKNOX3* expression domain (see **Fig 1B, 1G and 1L**). The expression of *MtIPT3* and *MtKNOX3* also demonstrate overlapping patterns in the vascular bundles of the developing nodules (12 dpi, **Fig 1C and 1H**) and partially in the apical part of the mature nodule. However, the *MtLOG2* promoter activity overlaps the *MtKNOX3* expression domain only at the early stage of nodule development (7 dpi), whereas at later stages when the *MtKNOX3* promoter activity shifts to the vascular bundles and to the apical part of the nodule, the *MtLOG2* expression retains in the central zone of the developing nodule, and later it is observed in the infection and the fixation zone, where no *MtKNOX3* promoter activity was detected. Thus, the expression patterns of the *MtKNOX3* gene and its putative targets partly overlap at some stages of nodule development, and this is in agreement with our suggestion that KNOX3 TF activates *MtLOG2* and *MtIPT3* expression in developing nodules. However, since *MtLOG2* and *MtKNOX3* expression patterns do not overlap at the later stages of nodule development we can not exclude that, in addition to MtKNOX3, other factors could be responsible for *MtLOG2* activation during nodulation. Otherwise, it can be speculated that MtKNOX3 transcription factor may act non-cell-autonomously, as it was shown for class I KNOX TFs [26]. Recently, symplastic communication was shown to be important for the formation of nitrogen-fixing nodules and, in particular, for the expression of the *NIN* (*NODULE INCEPTION*) gene as well as its downstream targets in the cortical cells during nodule development [27]. However, additional experiments are required to check if the MtKNOX3 TF is able to move through plasmodesmata.

Previously, the activation of the *AtIPT7* genes was found to be regulated by class I KNOX TFs, in particular by SHOOTMERISTEMLESS (STM) in *A*.*thaliana* [6, 7]. *STM* gene expresses in shoot apical meristem (SAM) [28]. However, the expression of STM target gene, *AtIPT7*, is absent in SAM according to promorter:GUS study [29], while very low *AtIPT7* expression level in SAM was found according to transcriptomic data [30]. In this connection, the lack of visible overlap between *MtKNOX3* and *MtLOG2* expression patterns at later stages of nodule development in developing nodules can be explained by the limitations of gene expression analysis by promoter:GUS assays (since this method does not assess the localization of gene transcripts directly, but instead allows to estimate the localization of gene promoter activity based on histochemical staining of GUS enzymatic activity, where the intensity of GUS staining may vary depending on the conditions of staining procedures), the more so according to LCMD-RNAseq data [19] the expression of *MtKNOX3*, *MtLOG2*, and *MtIPT3* genes overlaps in different zones of mature nodules (see **S1 Fig**).

The spatial expression of the *LOG*s and *IPT*s genes has been also studied in *L*. *japonicus* [10]. The promoter of the *LjLog4* gene was activated in cortical cells surrounding infection threads. At later stages, the expression of *LjLog4* was observed in the vascular tissue at the base of nodules according to promoter:GUS assay. The expression of other cytokinin biosynthesis genes, including *LjIpt3*, *LjIpt4*, and *LjLog4*, was also associated with vascular tissues within the nodules [10]. In contrast to our data on *LOG*s and *IPT*s expression in *M*. *truncatula*, a species that form indeterminate nodules possessing a persistent meristem, cytokinin biosynthesis genes expression has not been visualized in the apical part of *L*. *japonicus* nodules, which lack persistent meristematic activity. It could be speculated that the expression of cytokinin biosynthesis genes in the apical part of the nodule can be required for persistent meristematic activity; however, the additional data are needed to prove this suggestion.

Using two different approaches, EMSA and SPR-based protein-DNA assay, we found that KNOX3 homeodomain directly binds to the TGAC motif-containing regulatory sequences of the *MtLOG1*, *MtLOG2*, and *MtIPT3* genes. The fact that the control sequences containing the mutated TGAC motif (TCGC) were not bound by KNOX3 homeodomain indicates that TGAC motif is indeed necessary for such interaction, which brings KNOX3 TF together with class I KNOX I TFs known to bind to TGAC motif [16]. Moreover, such regulatory sequence with two TGAC motifs was found in the first intron of *MtLOG1* gene that is also involved in nodulation. According to our data, KNOX3 homeodomain is capable to interact with this sequence *in vitro*. Although we investigated the interaction of KNOX3 homeodomain with regulatory sequences of cytokinin biosynthesis genes only *in vitro*, and additional studies including ChIP assay are required to prove such interaction *in planta*, collectively, our data suggest that MtKNOX3 regulates nodule development through the direct activation of cytokinin biosynthesis genes, namely *MtLOG2* and *MtIPT3*, since the expression of these genes were decreased at *MtKNOX3*-RNAi background [9]. Our data partially contradict those of Di Giacomo et al. [11], who proposed a negative effect of MtKNAT3/4/5-like genes (including the *MtKNOX3* gene) on cytokinin signaling [11]. Similar to our results on MtKNOX3 RNAi, the simultaneous down-regulation of *MtKNAT*3/4/5-like genes resulted in the decreased expression of cytokinin response gene *MtRR4*. However, the expression of the *MtEFD* gene which had been previously shown to activate *MtRR4* expression [12] was also decreased, allowing the authors to suggest that *MtRR4* down-regulation could be explained by the decreased expression of the *MtEFD* gene in the transgenic roots with RNAi of the *MtKNAT*3/4/5-like genes. Being a primary cytokinin-activated gene, *MtRR4* as a member of *Response Regulator A* (*RRA*) gene family is also supposed to act as a negative regulator of cytokinin signaling [31], which allows the authors to suggest that *MtKNAT*3/4/5-like genes inhibit cytokinin signaling through EFD/RR4 regulatory module [11]. However, collectively, our data favor a hypothesis that the decreased expression of *MtRR4* in an MtKNOX3-RNAi background is due to the down-regulation of cytokinin biosynthesis genes, since the expression levels of the *MtIPT3* and *MtLOG2* genes were decreased in MtKNOX3-RNAi transgenic roots and consistently up-regulated in spontaneous nodule-like structures resulted from *MtKNOX3* overexpression [9], and the direct binding of MtKNOX3 TF with the regulatory sequences of these two genes strongly supports our hypothesis. Cytokinin was shown to play a complex role in nodulation, positively regulating nodule primordia formation and nitrogen fixation, while negatively regulating the rhizobial infection in the epidermis [32] and mediating negative feedback regulatory mechanisms of nodule development [25, 33]. Therefore, the MtKNOX3 TF, being an activator of cytokinin biosynthesis pathway, also plays a dual role in nodulation, both a positive and a negative one, depending on the time and the place of its action.

In the present study we demonstrated that class II KNOX TF is involved in KNOX-cytokinin regulatory module as it was previously shown for class I KNOX TF. Therefore, in contrast to other characterized members of class II KNOX TFs, KNOX3 plays a special role in nodule development, which places this regulator together with class I TFs playing a positive role in cell proliferation and meristem functioning through a cytokinin-mediated pathway.

## Supporting information

S1 Fig Expression of *MtKNOX3*, *MtIPT3*, and *MtLOG2* genes in different nodule zones according to LCM-RNA-seq data, publicly available on the INRA website (https://iant.toulouse.inra.fr/symbimics/). Meristematic zone (FI), distal and proximal infection zone (FIId and FIIp), inter-zone (IZ) and fixation zone (ZIII).
(PPTX)

S2 Fig Tissue specific expression of pIPT3::GUS in the lateral roots. Bars, 100 μm. Thickness of sections, 50 μm.
(TIF)

S3 Fig Homeodomain of MtKNOX3 transcription factor was synthesized in *P*. *pastoris* host. The results of protein electrophoresis of MtKNOX3 homeodomain (left) and western blot hybridization (right) with anti c-Myc antibody (Cat. No. 13–2500, Thermo Fisher Scientific, USA). 1- The protein after purification, 2- molecular weight marker (Cat. No. #26616, Thermo Fisher Scientific, USA).
(PDF)

S4 Fig Sensograms showing the interaction of the MtKNOX3 homeodomain with the regulatory sequences of MtLOG2 (A), MtIPT3 (B) and MtLOG1 (C) genes.
(JPG)

S5 Fig The result of SPR (A) and EMSA (B) for the negative control (poly A-T sequence).
(PDF)
