# Correction: Cytokinin biosynthesis genes expressed during nodule organogenesis are directly regulated by the KNOX3 protein in *Medicago truncatula*

**DOI:** 10.1371/journal.pone.0234022

**Published:** 2020-05-29

**Authors:** The PLOS ONE Staff

The corresponding author designation is not indicated in the article. The corresponding author is Maria A. Lebedeva. Maria A. Lebedeva’s email addresses are m.a.lebedeva@spbu.ru and mary_osipova@mail.ru.

The image for Fig 3 is incorrect. Please see the correct Fig 3 here.

**Fig 3:**
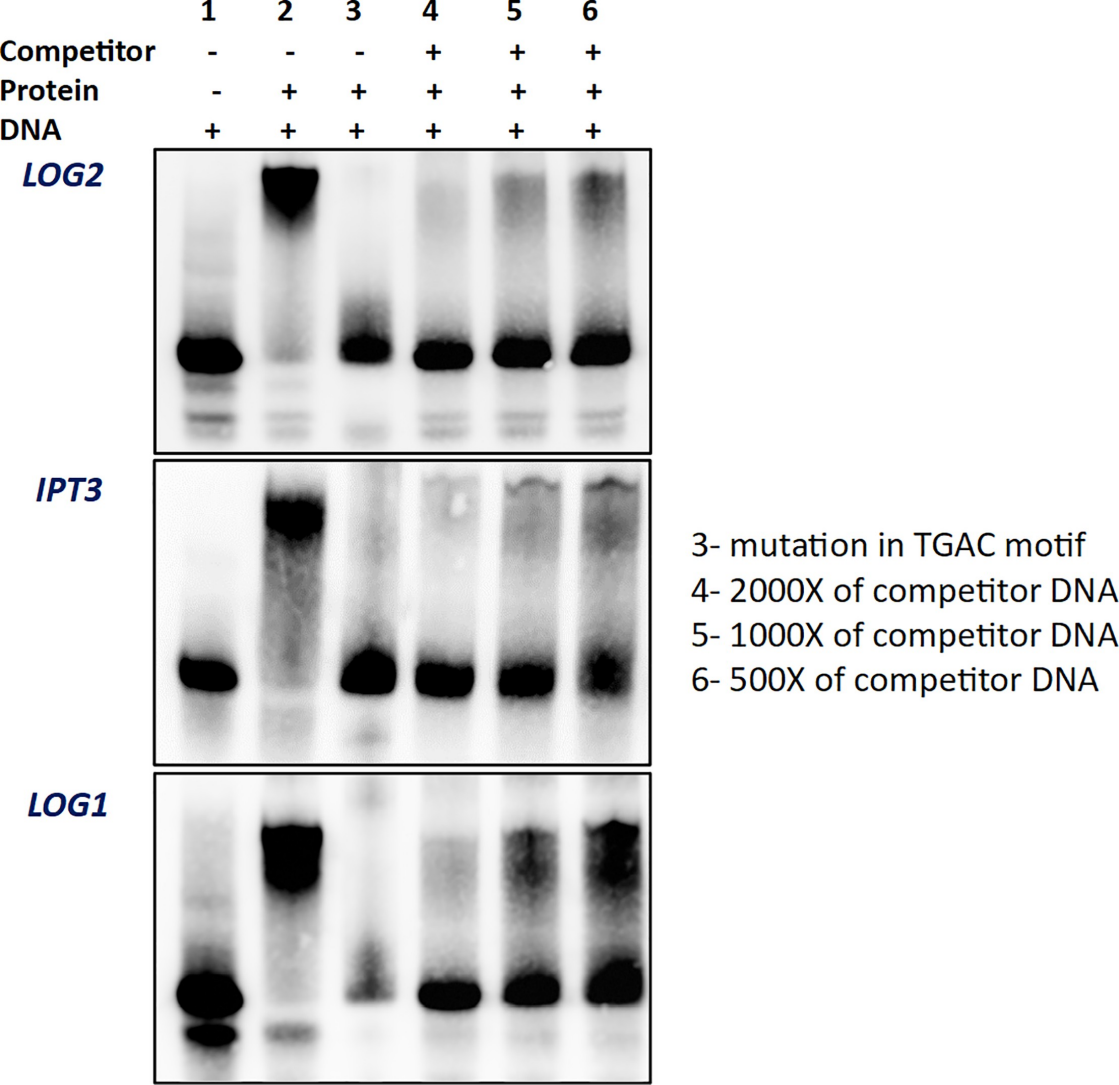
The results of EMSA experiment for the analysis of interaction between the homeodomain of MtKNOX3 and the regulatory sequences of the *MtLOG2*, *MtIPT3*, and *MtLOG1* genes. 1- free ds-DNA, 2- ds-DNA with the protein, 3- mutated ds-DNA with the protein, 4- ds-DNA with the protein and 2000X of competitor DNA, 5- ds-DNA with the protein and 1000X of competitor DNA, 6- ds-DNA with the protein and 500X of competitor DNA. The protein amount is the same in all wells (1000 ng).

The files for S3 Fig and S4 Fig are incorrectly switched. The file that appears as S3 Fig should be S4 Fig, and the file that appears as S4 Fig should be S3 Fig. The Supporting information captions appear in the correct order. Please see the correct files and captions here.

S1 File is omitted from the list of Supporting Information. It can be viewed here.

The publisher apologizes for the errors.

## Supporting information

S3 Fig Homeodomain of MtKNOX3 transcription factor was synthesized in *P*. *pastoris* host. The results of protein electrophoresis of MtKNOX3 homeodomain (left) and western blot hybridization (right) with anti c-Myc antibody (Cat. No. 13–2500, Thermo Fisher Scientific, USA). 1- The protein after purification, 2- molecular weight marker (Cat. No. #26616, Thermo Fisher Scientific, USA).
(JPG)

S4 Fig Sensograms showing the interaction of the MtKNOX3 homeodomain with the regulatory sequences of MtLOG2 (A), MtIPT3 (B) and MtLOG1 (C) genes.
(PDF)

S1 File Raw images. Unadjusted and uncropped gel/blot images underlying figures.
(PDF)
