# Supplementary figures and images for: Cytokinin biosynthesis genes expressed during nodule organogenesis are directly regulated by the KNOX3 protein in Medicago truncatula
Source: PLoS One. 2020 Apr 30;15(4):e0232352. doi: 10.1371/journal.pone.0232352 (PMC7192382; doi:10.1371/journal.pone.0232352)

## Slide 1
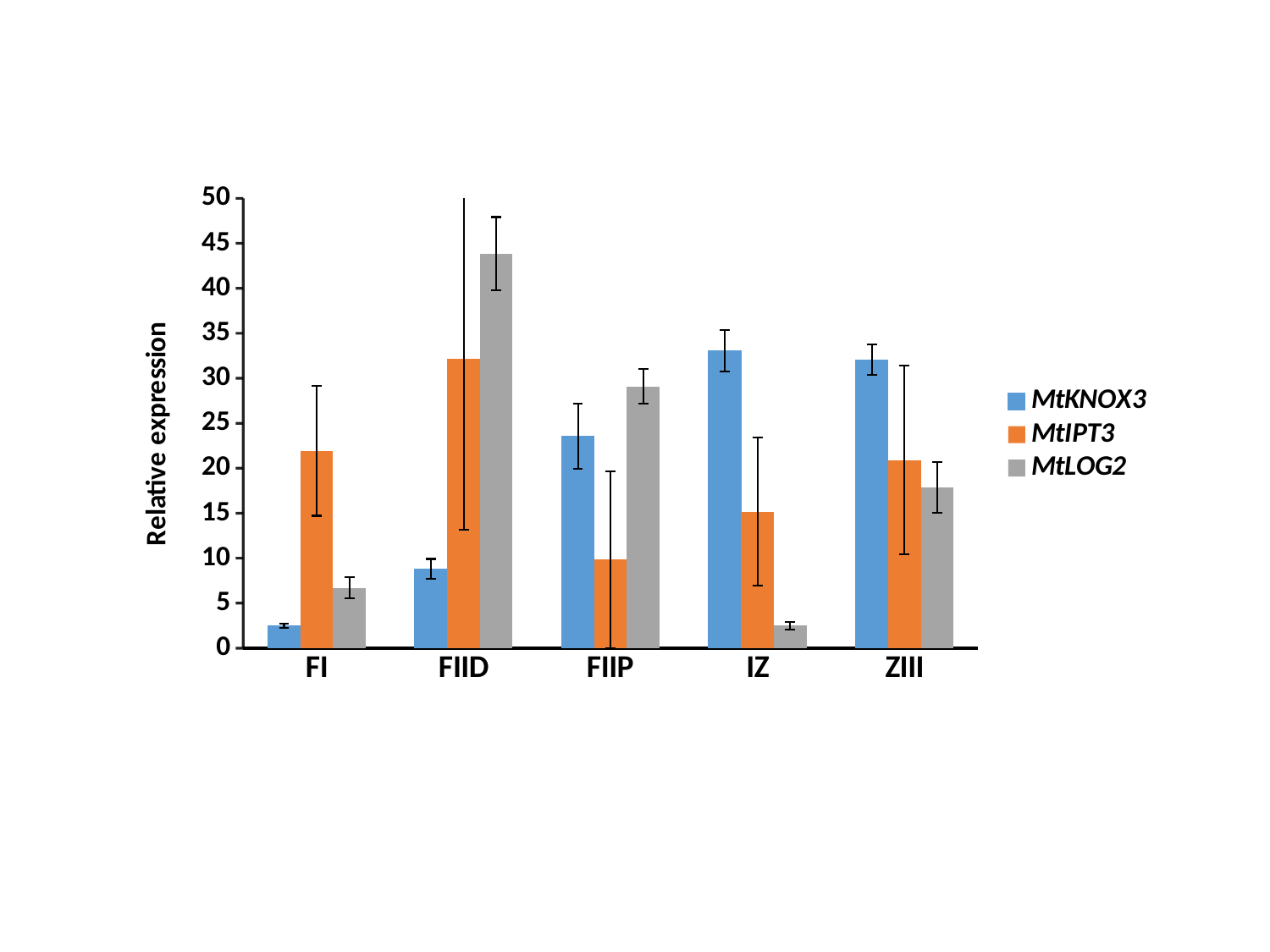

### Chart
| Category | MtKNOX3 | MtIPT3 | MtLOG2 |
|---|---|---|---|
| FI | 2.5 | 21.95 | 6.71 |
| FIID | 8.81 | 32.15 | 43.85 |
| FIIP | 23.55 | 9.83 | 29.08 |
| IZ | 33.08 | 15.15 | 2.5 |
| ZIII | 32.07 | 20.91 | 17.87 |

Supplement: S1 Fig — Meristematic zone (FI), distal and proximal infection zone (FIId and FIIp), inter-zone (IZ) and fixation zone (ZIII). (PPTX) [file pone.0232352.s001.pptx]

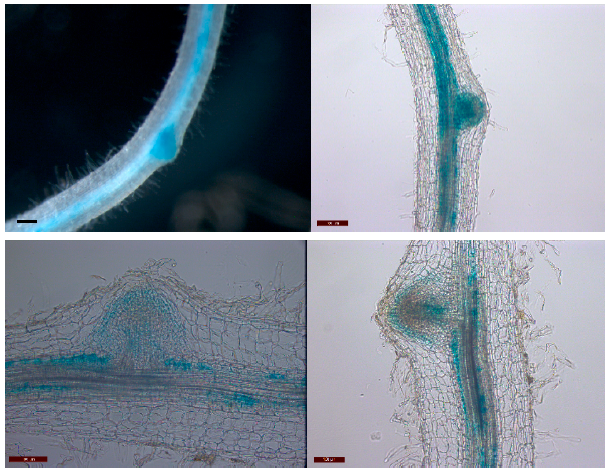

Supplement: S2 Fig — Bars, 100 μm. Thickness of sections, 50 μm. (TIF) [file pone.0232352.s002.tif]

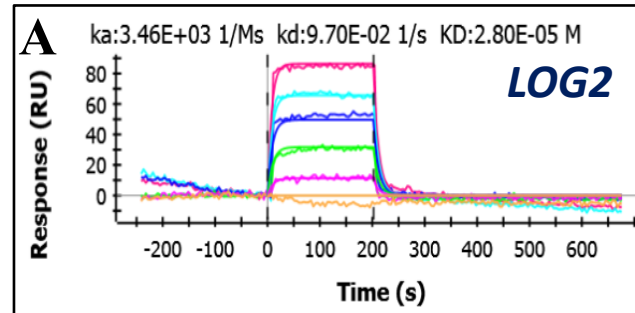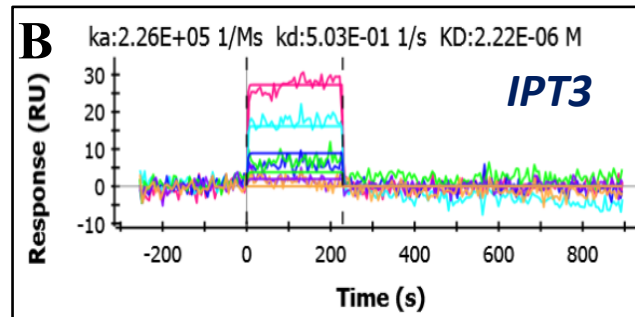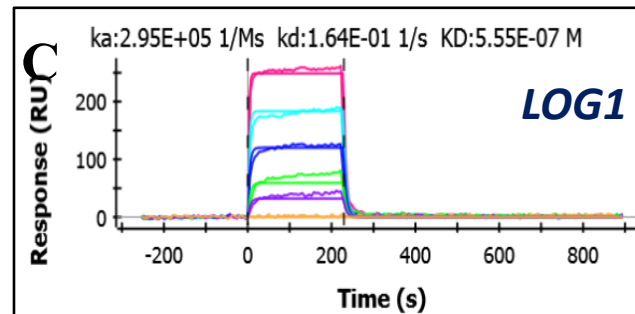

Supplement: S3 Fig — The results of protein electrophoresis of MtKNOX3 homeodomain (left) and western blot hybridization (right) with anti c-Myc antibody (Cat. No. 13–2500, Thermo Fisher Scientific, USA). 1- The protein after purification, 2- molecular weight marker (Cat. No. #26616, Thermo Fisher Scientific, USA). (PDF) [file pone.0232352.s003.pdf]

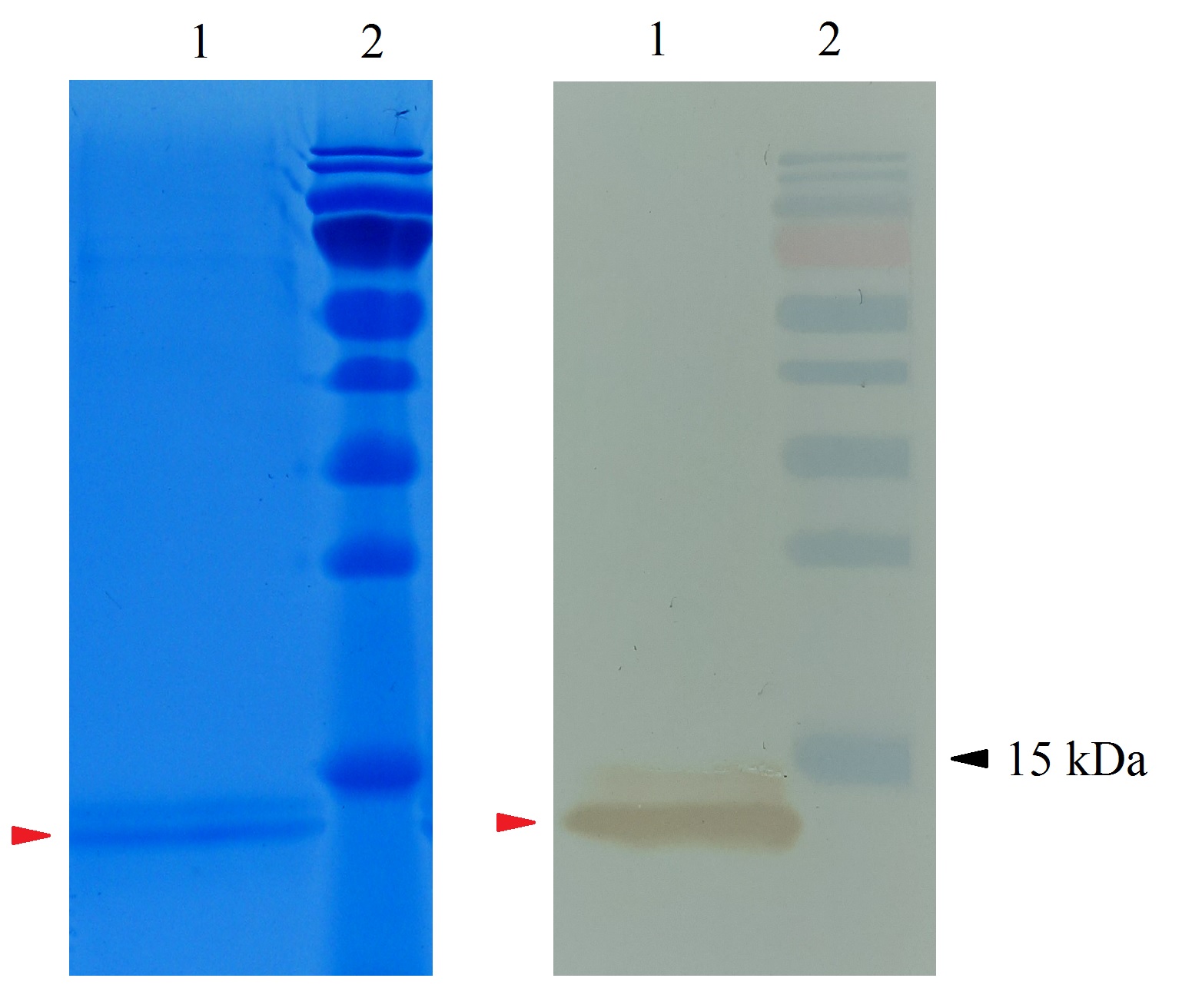

Supplement: S4 Fig — Sensograms showing the interaction of the MtKNOX3 homeodomain with the regulatory sequences of MtLOG2 (A), MtIPT3 (B) and MtLOG1 (C) genes. (JPG) [file pone.0232352.s004.jpg]

**A**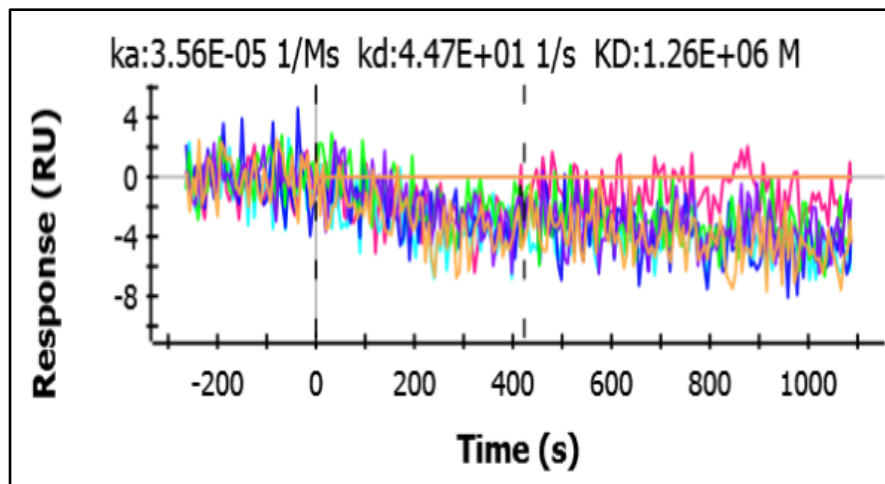**B**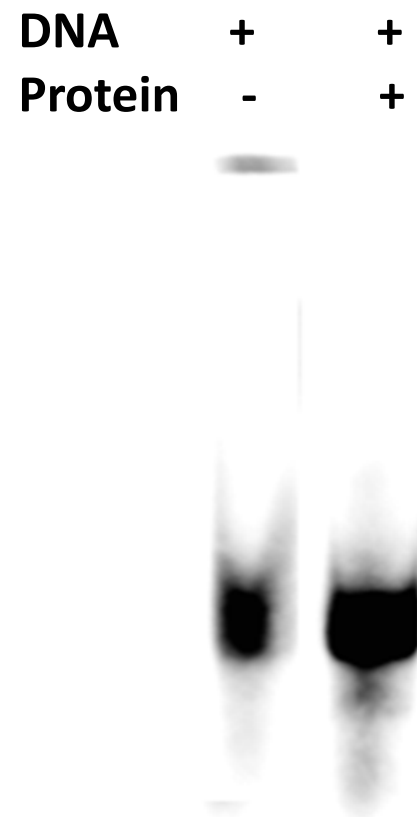

Supplement: S5 Fig — The result of SPR (A) and EMSA (B) for the negative control (poly A-T sequence). (PDF) [file pone.0232352.s005.pdf]
