# Supplementary figures and images for: Correction: Cytokinin biosynthesis genes expressed during nodule organogenesis are directly regulated by the KNOX3 protein in Medicago truncatula
Source: PLoS One. 2020 May 29;15(5):e0234022. doi: 10.1371/journal.pone.0234022 (PMC7259592; doi:10.1371/journal.pone.0234022)

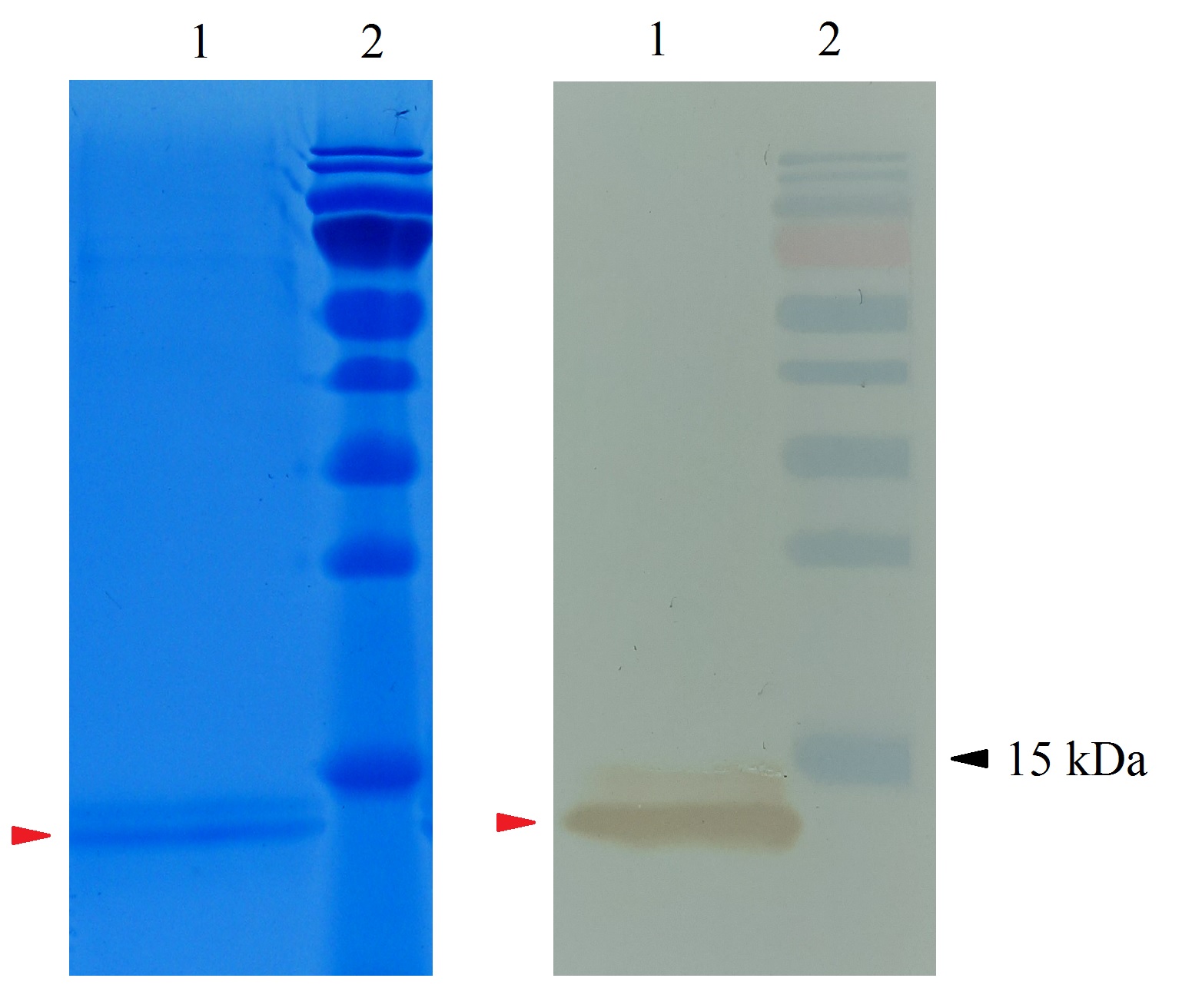

Supplement: S3 Fig — The results of protein electrophoresis of MtKNOX3 homeodomain (left) and western blot hybridization (right) with anti c-Myc antibody (Cat. No. 13–2500, Thermo Fisher Scientific, USA). 1- The protein after purification, 2- molecular weight marker (Cat. No. #26616, Thermo Fisher Scientific, USA). (JPG) [file pone.0234022.s001.jpg]

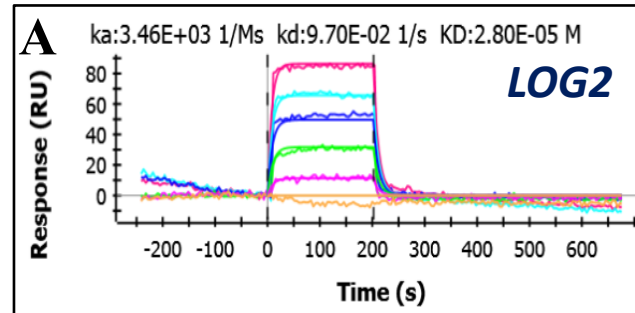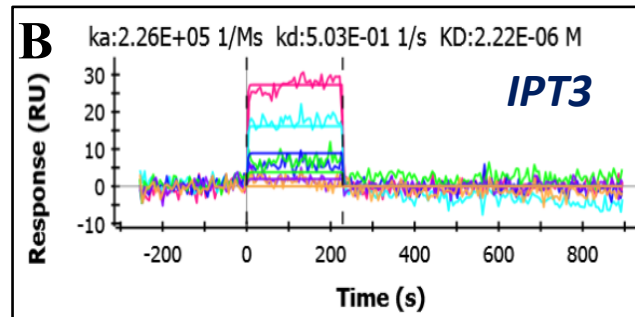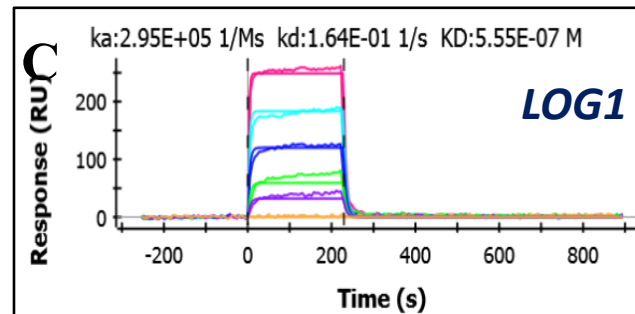

Supplement: S4 Fig — Sensograms showing the interaction of the MtKNOX3 homeodomain with the regulatory sequences of MtLOG2 (A), MtIPT3 (B) and MtLOG1 (C) genes. (PDF) [file pone.0234022.s002.pdf]
