# Supplementary material for: Correction: Cytokinin biosynthesis genes expressed during nodule organogenesis are directly regulated by the KNOX3 protein in Medicago truncatula
Source: PLoS One. 2020 May 29;15(5):e0234022. doi: 10.1371/journal.pone.0234022 (PMC7259592; doi:10.1371/journal.pone.0234022)

Raw images for Figure 3:

Upper panel (LOG2)
Middle panel (IPT3)
Lower panel (LOG1)

The raw images for the EMSA experiment for the analysis of interaction between the homeodomain of MtKNOX3 and the regulatory sequences of the MtLOG2, MtIPT3, and MtLOG1 genes. Biotinylated DNA on the membrane was detected by chemiluminescence (Chemiluminescent Nucleic Acid Detection Module Kit, Thermo Scientific, USA) using GeneGnome XRQ - Chemiluminescence imaging system (SynGene, India). For each panel the original and the inverted images are presented.

The labeling of the lanes is the same as in Figure 2:

1- free ds-DNA,
2- ds-DNA with the protein,
3- mutated ds-DNA with the protein,
4- ds-DNA with the protein and 2000X of competitor DNA,
5- ds-DNA with the protein and 1000X of competitor DNA,
6- ds-DNA with the protein and 500X of competitor DNA.

The protein amount is the same in all wells (1000 ng).

Figure 3, Upper panel (LOG2)

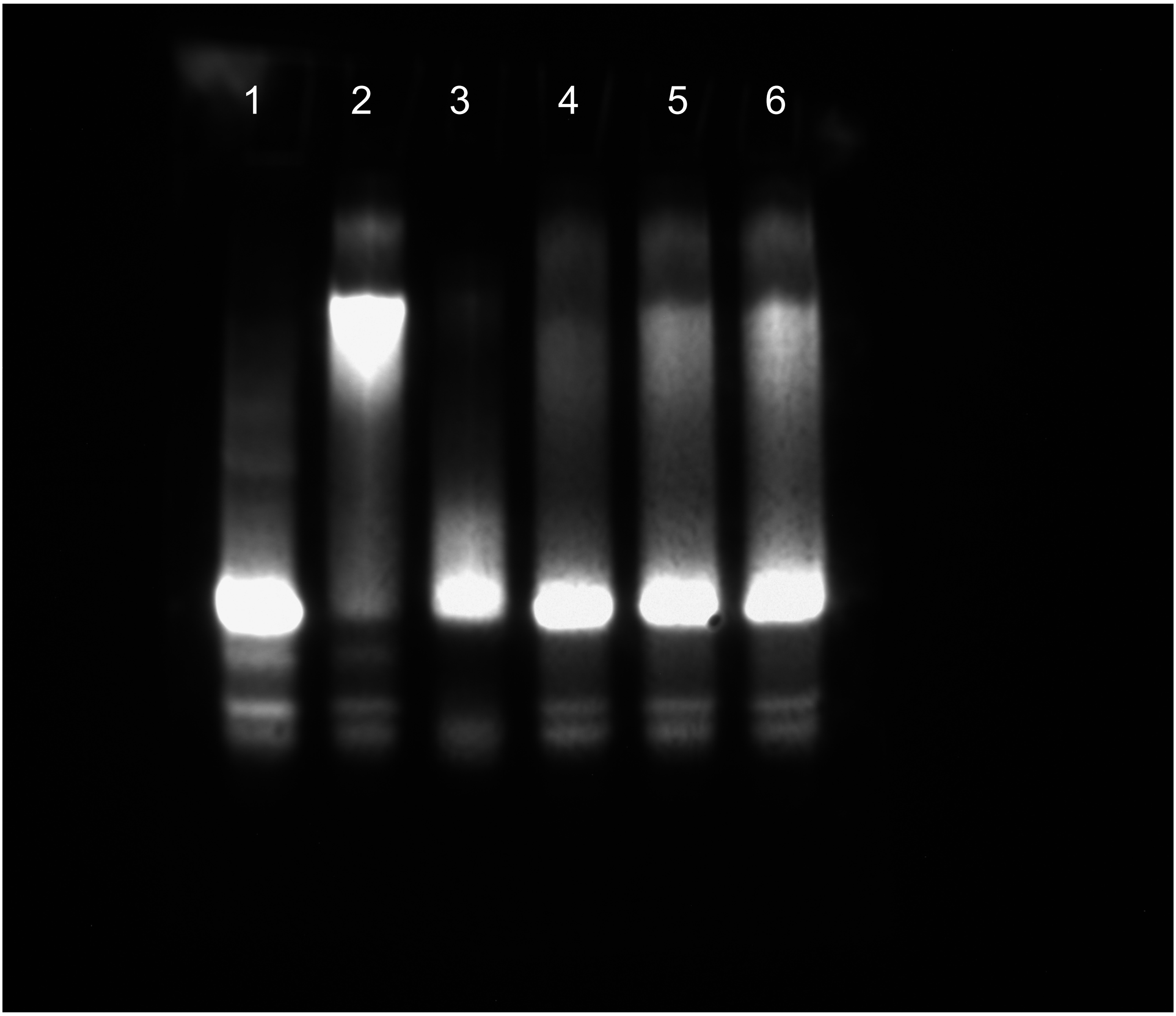

Figure 3, Middle panel (IPT3)

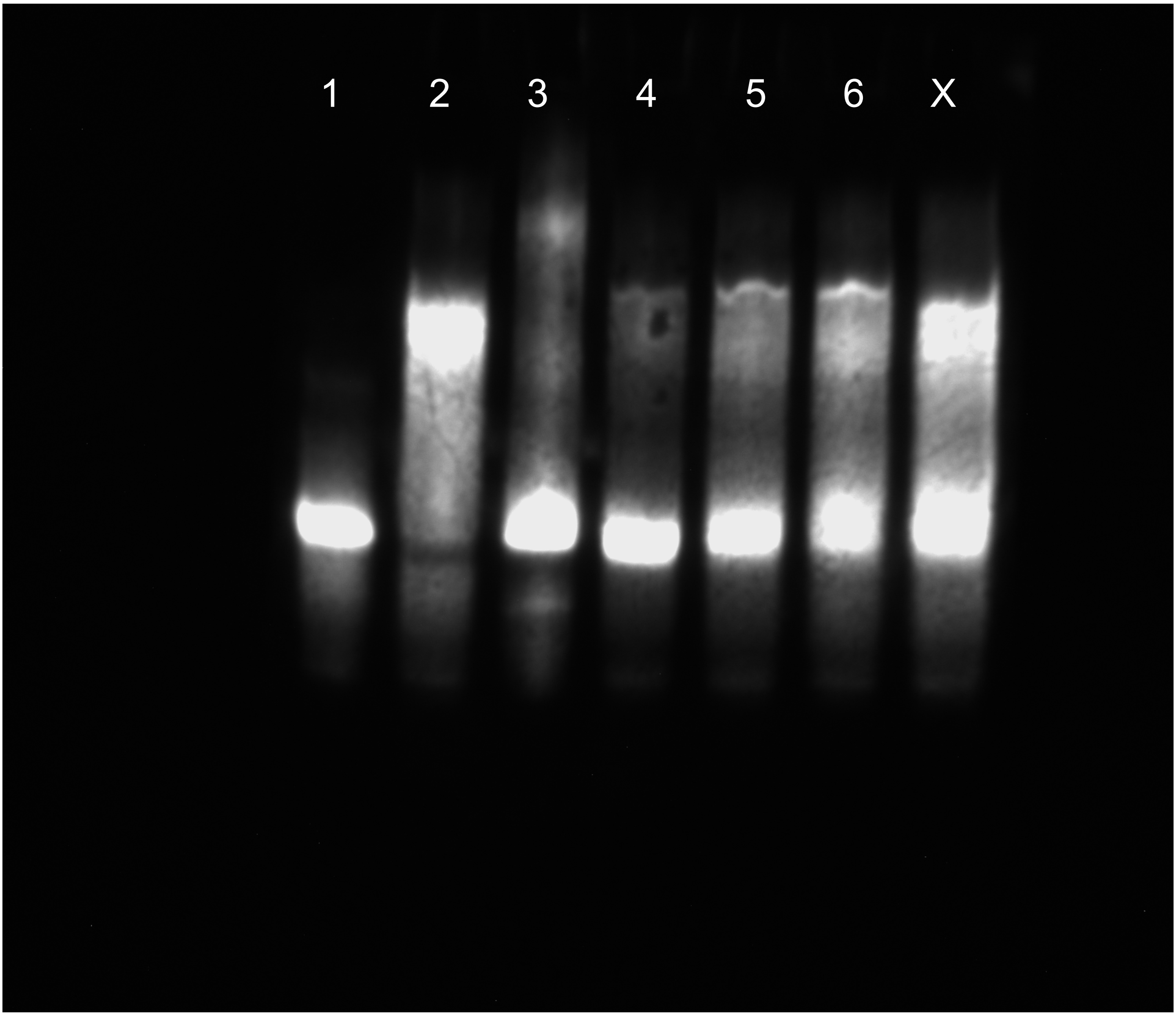

Figure 3, Lower panel (LOG1)

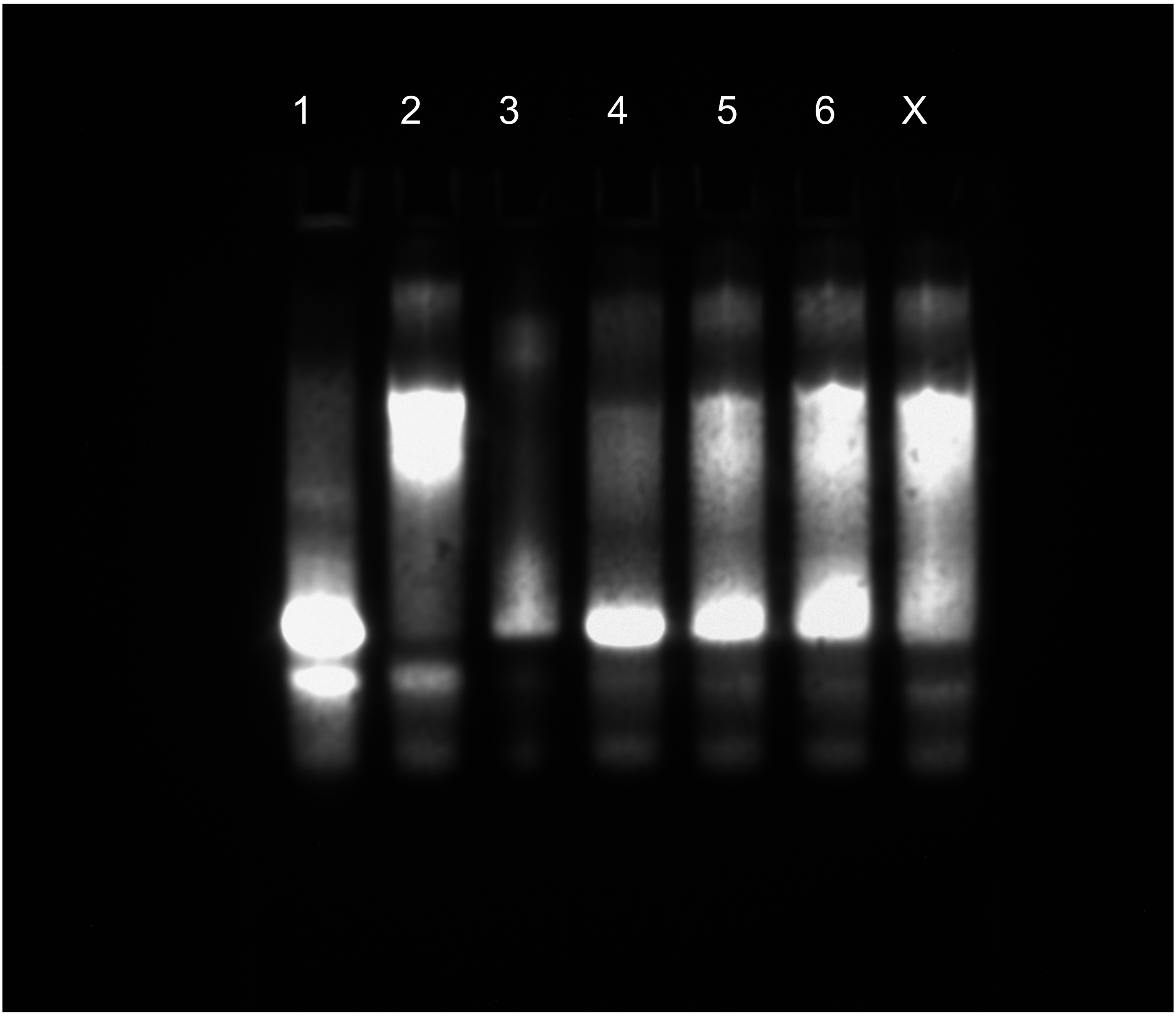

Raw images for Supplementary Figure 3:

The results of protein electrophoresis of MtKNOX3 homeodomain (left panel in Supplementary Figure 3)

1- The protein after purification,
2- molecular weight marker (Cat. No. #26616, Thermo Fisher Scientific, USA).

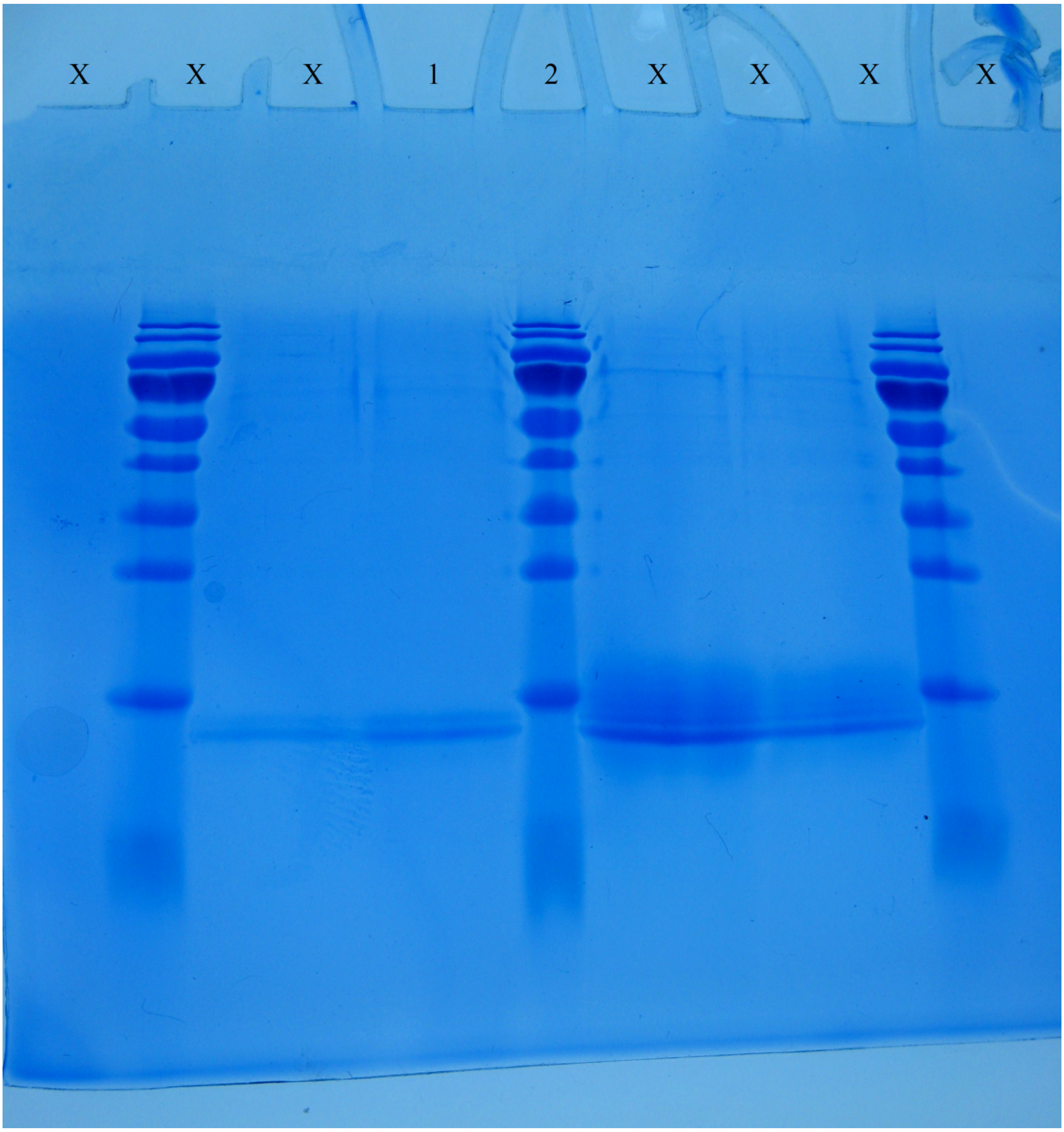

Raw images for Supplementary Figure 3:

The results of western blot hybridization of purified MtKNOX3 homeodomain (right panel in Supplementary Figure 3) with anti c-Myc antibody (Cat. No. 13-2500, Thermo Fisher Scientific, USA).

1- The protein after purification,
2- molecular weight marker (Cat. No. #26616, Thermo Fisher Scientific, USA).

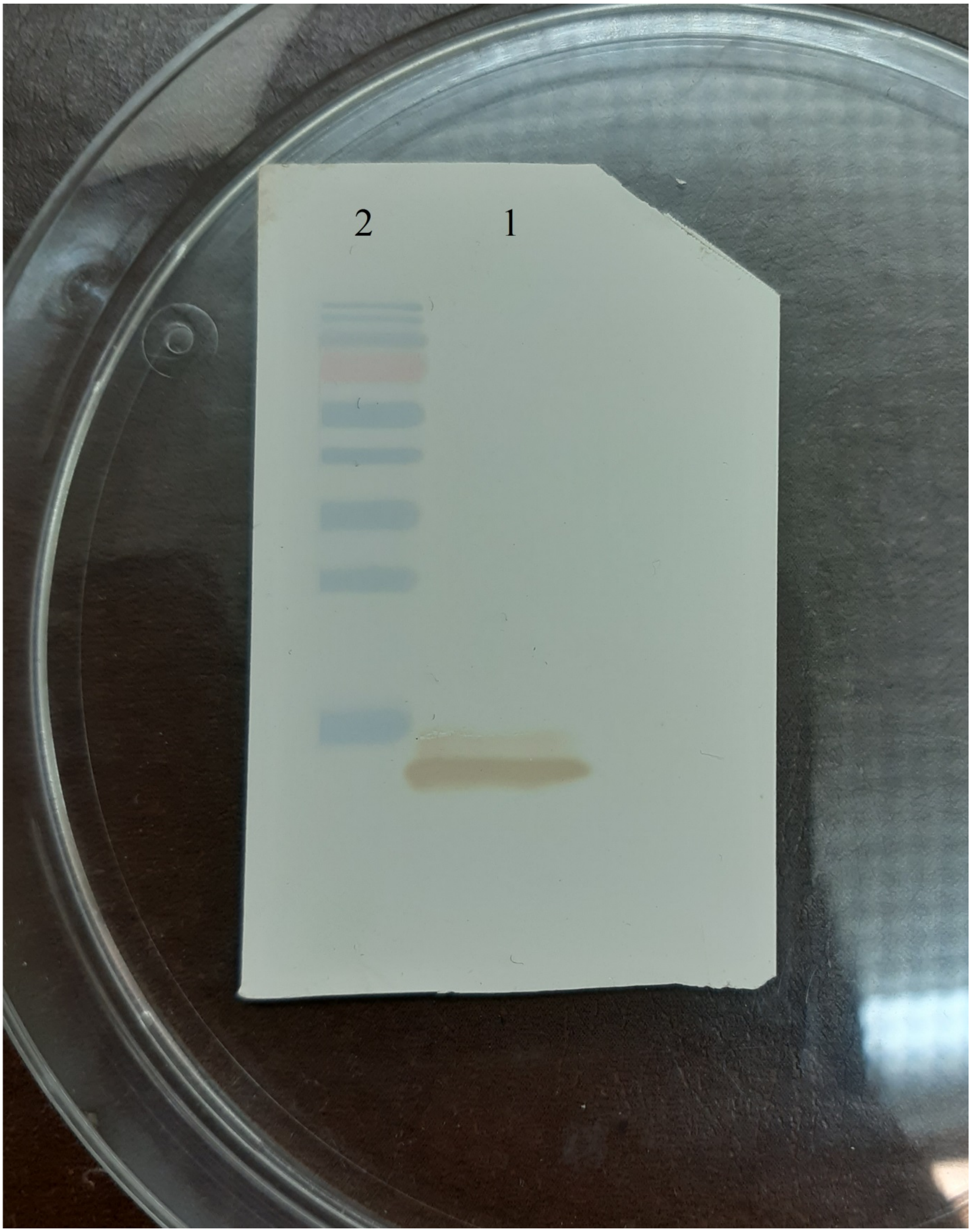

Raw image for Supplementary Figure 5, Panel B:

The results of EMSA for the negative control (poly A-T sequence).

1- free DNA
2- DNA+protein

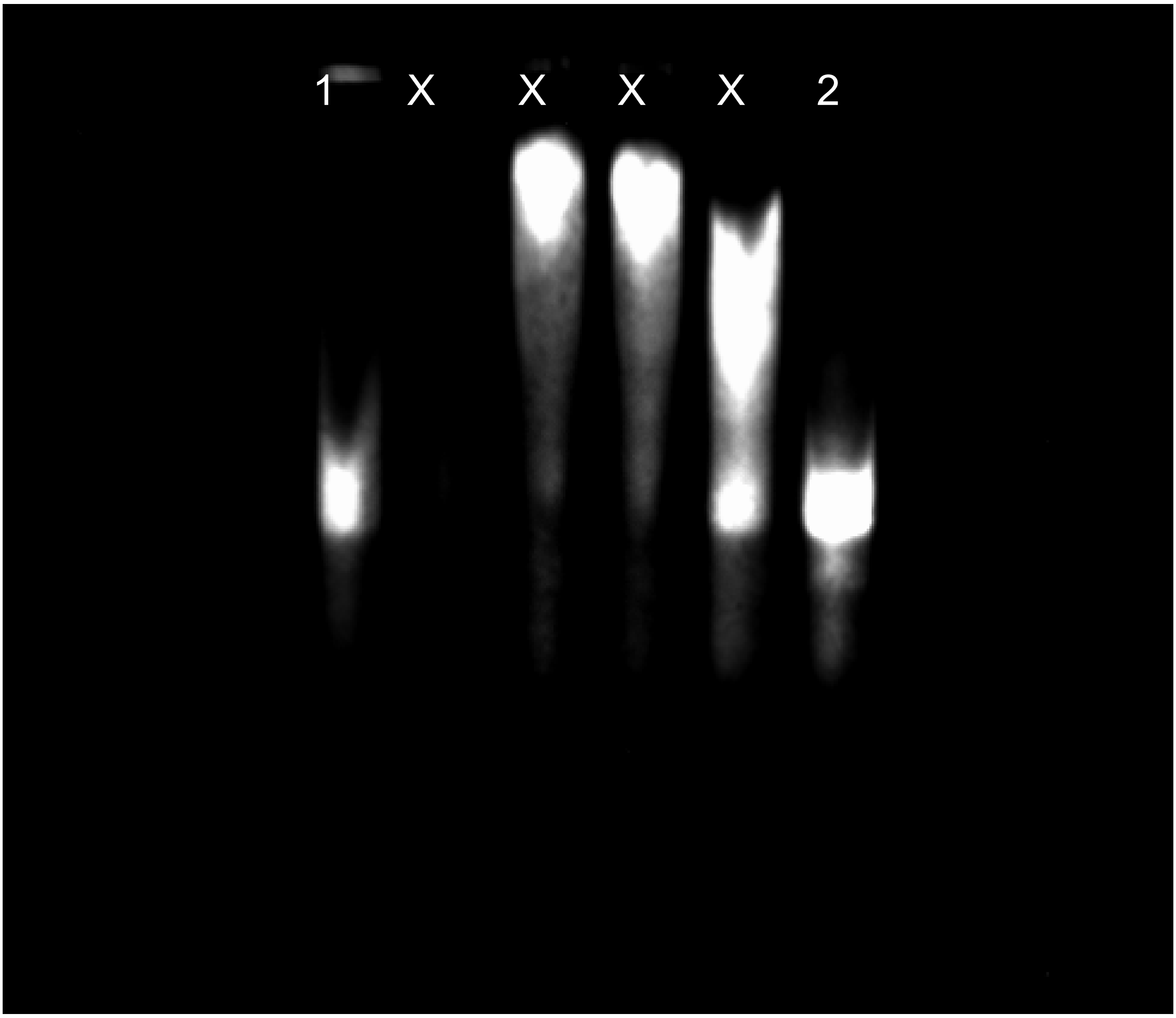

Supplement: S1 File — Unadjusted and uncropped gel/blot images underlying figures. (PDF) [file pone.0234022.s003.pdf]
